# Enhanced Performance of Close‐Spaced Sublimation Processed Antimony Sulfide Solar Cells via Seed‐Mediated Growth

**DOI:** 10.1002/advs.202409312

**Published:** 2024-10-21

**Authors:** Wentao Wu, Bo Tang, Lei Wan, Xiaoli Mao, Haolin Wang, Guoqing Tong, Tao Chen, Ru Zhou

**Affiliations:** ^1^ School of Electrical Engineering and Automation Hefei University of Technology Hefei 230009 P. R. China; ^2^ School of Physics Hefei University of Technology Hefei 230009 P. R. China; ^3^ Hefei National Research Center for Physical Sciences at the Microscale School of Chemistry and Materials Science University of Science and Technology of China Hefei 230026 P. R. China; ^4^ School of Materials Science and Engineering Hefei University of Technology Hefei 230009 P. R. China

**Keywords:** close‐spaced sublimation, open‐circuit voltage, seed‐mediated growth, seed layers, Sb_2_S_3_ solar cells

## Abstract

Antimony sulfide (Sb_2_S_3_) has attracted much attention due to its great prospect to construct highly efficient, cost‐effective, and environment‐friendly solar cells. The scalable close‐spaced sublimation (CSS) is a well‐developed physical deposition method to fabricate thin films for photovoltaics. However, the CSS‐processed absorber films typically involve small grain size with high‐density grain boundaries (GBs), resulting in severe defects‐induced charge‐carrier nonradiative recombination and further large open‐circuit voltage (*V*_OC_) losses. In this work, it is demonstrated that a chemical bath deposited‐Sb_2_S_3_ seed layer can serve as crystal nuclei and mediate the growth of large‐grained, highly compact CSS‐processed Sb_2_S_3_ films. This seed‐mediated Sb_2_S_3_ film affords reduced defect density and enhanced charge‐carrier transport, which yields an improved power conversion efficiency (PCE) of 4.78% for planar Sb_2_S_3_ solar cells. Moreover, the *V*_OC_ of 0.755 V that is obtained is the highest reported thus far for vacuum‐based evaporation and sublimation processed Sb_2_S_3_ devices. This work demonstrates an effective strategy to deposit high‐quality low‐defect‐density Sb_2_S_3_ films via vacuum‐based physical methods for optoelectronic applications.

## Graphical abstract

A seed‐mediated close‐spaced sublimation strategy is developed to grow large‐grained and highly compact Sb_2_S_3_ thin films, which afford reduced defect density and enhanced charge‐carrier transport. An enhanced power conversion efficiency of 4.78% is obtained for planar Sb_2_S_3_ solar cells. A high *V*_OC_ of 0.755 V is the record value reported thus far for vacuum‐based evaporation and sublimation‐processed Sb_2_S_3_ devices.

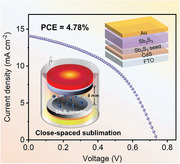

## 1 Introduction

Thin‐film solar cells play a great role in building integrated photovoltaics (BIPV), space industry, indoor photovoltaics (IPVs), etc.^[^ ^1^, ^2^, ^3^, ^4^ ^]^ The ongoing exploration of highly efficient, cost‐effective, and environment‐friendly solar cells has led to the emergence of new light‐harvesting materials for thin‐film photovoltaics. Quasi‐one‐dimensional (quasi‐1D) antimony chalcogenides, mainly including Sb_2_S_3_, Sb_2_Se_3_, and ternary alloyed Sb_2_(S,Se)_3_, have received increasing attention in view of their excellent materials and optoelectronic properties, including simple composition, earth‐abundance, excellent stability, high absorption coefficients, efficient charge transport along covalently bonded [Sb_4_S_6_]_n_ ribbons, etc.^[^ ^5^, ^6^, ^7^, ^8^ ^]^ In the past decade, the power conversion efficiencies (PCEs) of planar‐type antimony chalcogenide solar cells have made great advances, exceeding a landmark value of 10% for potential commercial photovoltaic application.^[^ ^9^, ^10^, ^11^ ^]^ The binary Sb_2_S_3_ has an appropriate bandgap of ≈1.7 eV and a large absorption coefficient of 10^4^–10^5^ cm^−1^ over the UV‐visible range, enabling it to be an ideal candidate for IPVs as well as top cells of silicon‐based tandem solar cells.^[^ ^12^, ^13^, ^14^, ^15^ ^]^

The close‐spaced sublimation (CSS) method is a well‐established film deposition approach for semiconductor technologies. A representative case is the use of CSS to fabricate commercial CdTe thin‐film solar cells.^[^ ^16^ ^]^ Following the efforts of researchers working on antimony chalcogenides, CSS has also been successfully developed to deposit high‐quality Sb_2_S_3_, Sb_2_Se_3_, and Sb_2_(S,Se)_3_ thin films.^[^ ^17^, ^18^, ^19^ ^]^ Compared to other chemical solution‐based methods (spin‐coating, hydrothermal, chemical bath deposition (CBD), etc.), this vacuum deposition technique is capable of realizing high‐throughput formation of large‐area, compact and uniform films.^[^ ^12^, ^13^ ^]^ In addition, Sb_2_S_3_ has a low melting point of ≈550 °C and a high saturation vapor pressure, enabling the facile deposition of Sb_2_S_3_ films under low temperatures.^[^ ^20^ ^]^ For instance, Krautmann et al. obtained crack‐free Sb_2_S_3_ films with the presence of (hk1) planes via CSS under a low substrate temperature of 300 °C, delivering a PCE of 3.8%.^[^ ^21^ ^]^ Guo et al. achieved a PCE of 3.83% with the configuration of glass/FTO/CdS/Sb_2_S_3_/graphite back contact by a scalable CSS and revealed that Sb_2_S_3_ showed intrinsic p‐type owing to S_Sb_ antisites and the device performance was limited by the presence of S vacancies.^[^ ^17^ ^]^ To date, although interesting achievements have been made on CSS‐processed Sb_2_S_3_ solar cells, the corresponding device efficiencies still lag far behind those of the devices prepared by chemical solution methods, which have pushed the PCE of Sb_2_S_3_ devices up to 8%.^[^ ^22^, ^23^ ^]^ Moreover, according to the detailed balance limit for single p‐n junctions, the theoretical maximum device performance parameters for the device with a 1.70 eV bandgap under one‐sun AM 1.5G illumination are an open‐circuit voltage (*V*_OC_) of 1.402 V, a short‐circuit current density (*J*_SC_) of 22.46 mA cm^−2^, a fill factor (FF) of 91%, and a PCE of 28.64%.^[^ ^24^ ^]^ This indicates the great potential of further improvements for CSS‐processed Sb_2_S_3_ devices, which currently deliver the highest *V*_OC_ and PCE to be only 0.71 V and 3.83%, respectively.^[^ ^25^, ^26^ ^]^

One of the main concerns for CSS‐processed Sb_2_S_3_ solar cells lies with the severe defects‐induced charge‐carrier nonradiative recombination at the grain boundaries (GBs) and interfaces in the bulk absorber films, leading to large *V*_OC_ losses and relatively low PCEs.^[^ ^5^, ^27^ ^]^ It is known that, for polycrystalline semiconductor film‐based optoelectronic devices, GBs have significant impacts on the optoelectronic properties of the films, and ultimately on the performance of the devices.^[^ ^28^, ^29^, ^30^ ^]^ This is because GBs involving dangling or wrong bonds would impede the charge‐carrier transport, thereby increasing the rate of non‐radiative recombination.^[^ ^31^ ^]^ In addition, GBs also affect the film properties, including carrier mobility, conductivity, dielectric constant, etc.^[^ ^32^ ^]^ It is generally accepted to fabricate large‐grained absorber films for enhanced performance. For example, the engineering of Cu‐deficient GBs for Cu(In,Ga)Se_2_ (CIGS) films and CdCl_2_‐activation for CdTe films have been successfully explored to enhance the device's performance.^[^ ^33^, ^34^ ^]^ For widely studied organic‐inorganic halide perovskite solar cells, the deposition of perovskite films comprising large grains with ultralow‐density GB network also contributes to improved PCE and environmental stability.^[^ ^35^ ^]^ In view of the polycrystalline nature of Sb_2_S_3_ absorbers,^[^ ^36^ ^]^ it is of great potential to alleviate the charge‐carrier recombination loss issue for Sb_2_S_3_ devices if large‐grain spanning absorbers can be obtained. However, at present, the Sb_2_S_3_ films prepared by the physical deposition methods, including CSS, rapid thermal evaporation (RTE), and vapor transport deposition (VTD), typically exhibit smaller grain sizes compared to the commonly used solution‐processed films.^[^ ^17^, ^37^, ^38^ ^]^ Hence, achieving large grains of Sb_2_S_3_ thin films is of great importance to eliminate or reduce the detrimental impacts of GBs.

In this work, we incorporated a thin Sb_2_S_3_ seed layer on the buffer layer to mediate the subsequent growth of the CSS‐processed Sb_2_S_3_ absorber layer. This strategy helps to obtain high‐quality compact Sb_2_S_3_ thin films with reduced GBs. The increase in the grain size leads to a decrease in defect density, which suppresses the nonradiative charge‐carrier recombination in Sb_2_S_3_ films. The resulting planar Sb_2_S_3_ solar cell based on the seed‐mediated CSS‐processed Sb_2_S_3_ thin films yields enhanced PCE and *V*_OC_, mainly attributed to the suppression of nonradiative recombination as well as the improvement of heterointerface quality. This work demonstrates an effective seed‐mediation strategy to regulate the GBs of CSS‐deposited Sb_2_S_3_ films, which is beneficial to the performance enhancement of Sb_2_S_3_ solar cells.

## 2 Results and Discussion

**Figure 1:**
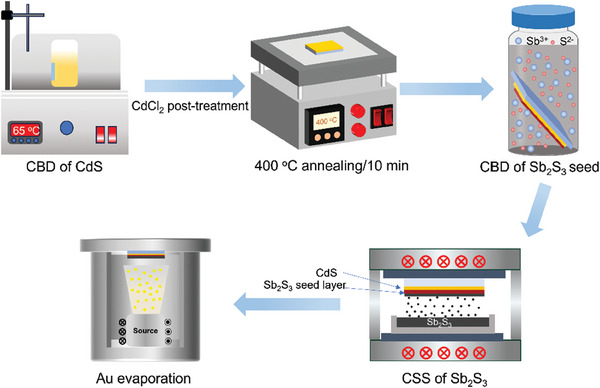
Schematic illustration of the device fabrication processes for planar Sb_2_S_3_ solar cells.

In this work, a simple superstrate device configuration (FTO/CdS/Sb_2_S_3_/Au) was used to construct planar Sb_2_S_3_ solar cells. The device fabrication processes for planar Sb_2_S_3_ solar cells are schematically illustrated in **Figure** **1**. The Sb_2_S_3_ seed layer was deposited on the FTO/CdS substrate via CBD, and the main Sb_2_S_3_ absorber layer was deposited using CSS. For more experimental details, please refer to the Experimental Section.

**Figure 2:**
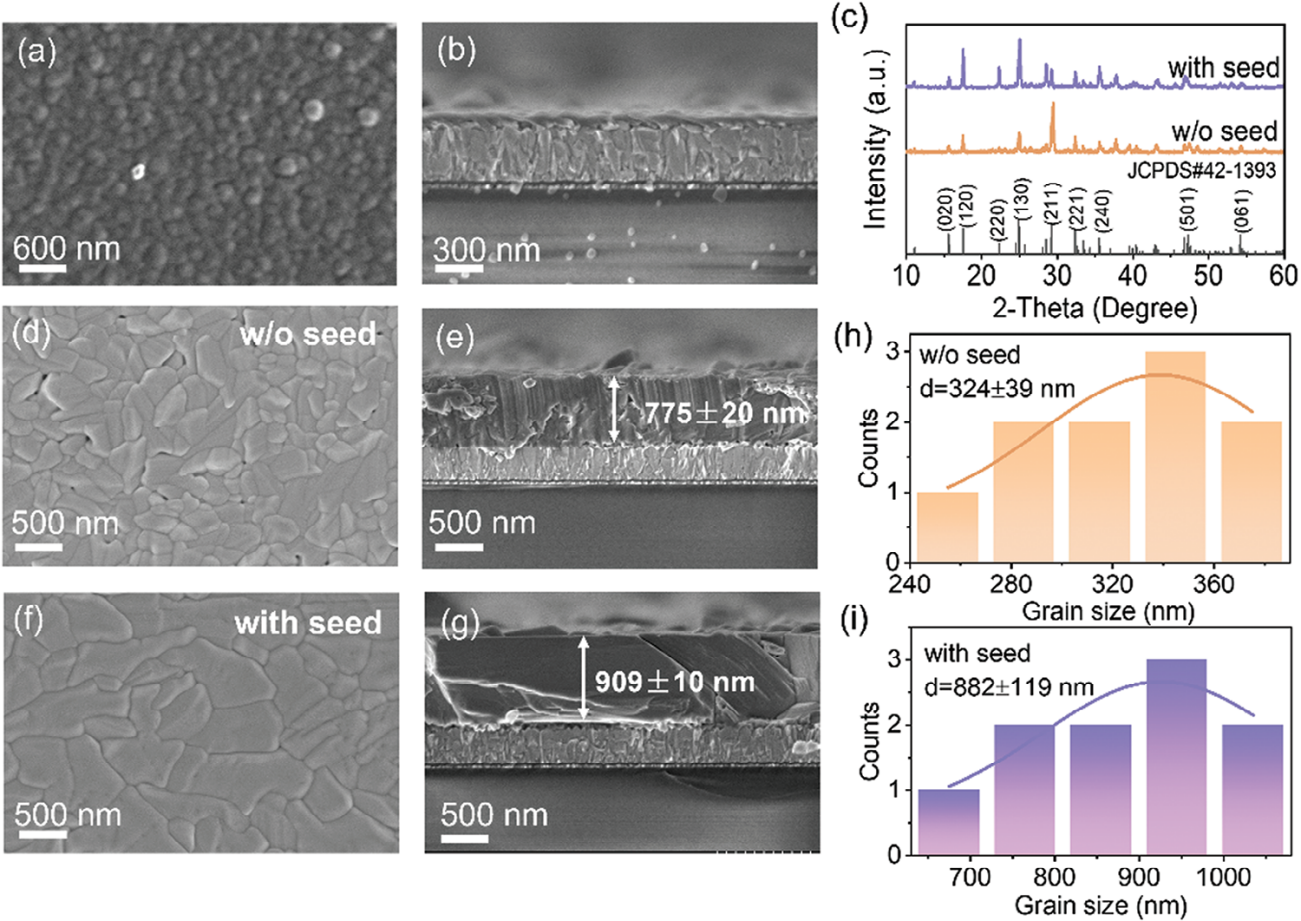
a) Top‐view and b) cross‐sectional‐view SEM images of the Sb_2_S_3_ seed layer. c) XRD patterns, d,f) Top‐view, and e,g) cross‐sectional‐view SEM images of Sb_2_S_3_ films prepared without and with seed. h,i) Statistics of grain sizes for the “w/o seed” (part d) and “with seed” (part f) film samples.

The top‐view and cross‐sectional scanning electron microscopy (SEM) images of the Sb_2_S_3_ seed layer deposited by CBD without annealing are shown in **Figure** **2**a,b. This seed layer exhibits uniform, compact morphology with a thickness of ≈ 70 nm on top of the CdS buffer layer. In addition, there are no distinct GBs on the film surface, implying the amorphous feature of the as‐obtained Sb_2_S_3_ seed layer. This is consistent with the X‐ray diffraction (XRD) pattern of the seed layer (Figure S1, Supporting Information), which only reveals distinct peaks for the FTO/CdS substrate. The main Sb_2_S_3_ absorber layer was further deposited onto the seed layer by using CSS. Herein, for the convenience of description, the CSS‐processed Sb_2_S_3_ absorber films prepared without and with the Sb_2_S_3_ seed layer are labeled as “w/o seed” and “with seed”. Figure 2c presents the XRD patterns of Sb_2_S_3_ films prepared with and without the seed layer. It is found that all the film samples exhibit a typical orthorhombic crystal structure of the Pbnm space group for Sb_2_S_3_ (JCPDS＃42‐1393).^[^ ^5^ ^]^ The dominating diffraction peaks, including (120), (130), (211), and (221) reflections, can be observed without noticeable impurities. The differences between both XRD patterns lie in the peak intensity, which indicates that the incorporation of the seed layer causes the change in the crystal orientation of Sb_2_S_3_ films. We further used the texture coefficients (TC) to evaluate the film orientation of Sb_2_S_3_ films (Figure S2, Supporting Information).^[^ ^5^ ^]^ It can be seen that the incorporation of the seed layer results in more pronounced [hk0] orientations for Sb_2_S_3_ films, such as [120] and [130]. Figure 2d,f shows top‐view SEM images of Sb_2_S_3_ films prepared without and with the seed layer, respectively. The “w/o seed” sample exhibits a compact morphology with some pinholes on the film surface, similar to the typical CSS‐deposited Sb_2_S_3_ films.^[^ ^21^ ^]^ By comparison, the film surface of the “with seed” sample becomes more compact, without obvious pinholes observed. Meanwhile, the grain size of the Sb_2_S_3_ thin film is significantly increased with the presence of the seed layer, as reflected by the statistical analysis of the grain size for both films (Figure 2h,i). The average grain size more than doubles from 324 ± 39 nm for the “w/o seed” sample to 882 ± 119 nm for the “with seed” sample. The cross‐sectional view SEM images (Figure 2e,g) reveal the film thickness of 775 ± 20 and 909 ± 10 nm for the “w/o seed” sample and the “with seed” sample, respectively. This indicates that the underlying seed layer not only greatly impacts the morphology of Sb_2_S_3_ films but also slightly influences the growth rate of films, with the small thickness of the seed layer taken into consideration.

Based on these results, it is obvious that the orientation and morphology of CSS‐Sb_2_S_3_ films are significantly affected by the Sb_2_S_3_ seed layer. We propose that the seed layer plays a crucial role in mediating the growth of subsequently deposited Sb_2_S_3_ films. For the conventional CSS deposition of Sb_2_S_3_ films directly on the CdS surface, the large lattice mismatch between CdS and Sb_2_S_3_ would result in a large interface energy between CdS and Sb_2_S_3_, which makes it difficult for Sb_2_S_3_ to nucleate on the CdS surface. The sublimated Sb_2_S_3_ particles tend to grow in the form of islands, which is detrimental to the formation of compact films.^[^ ^5^, ^38^ ^]^ In comparison, as we know, the CBD‐Sb_2_S_3_ typically involves large‐grained compact thin films with [hk0]‐dominated orientation on the CdS substrate.^[^ ^5^ ^]^ In this work, the CBD‐Sb_2_S_3_ seed layer affords a high density of nucleation sites for the subsequent CSS of Sb_2_S_3_. During this stage, the high‐density small Sb_2_S_3_ nuclei formed on the seed substrate would be more inclined to grow horizontally compared to the case without the seed layer, and thus further coalesce into a large‐grained compact Sb_2_S_3_ film. This scenario is similar to our previous work, which demonstrates that the generation of an ultrathin Ce_2_S_3_ interlayer at the CdS/Sb_2_S_3_ interface would facilitate the heterogeneous nucleation and the horizontal growth of Sb_2_S_3_ grains as a result of reduced nucleation‐free energy barrier and interfacial energy.^[^ ^39^ ^]^ Here, another possibility is that the sublimated Sb_2_S_3_ might involve the epitaxial growth upon the Sb_2_S_3_ seed layer, which results in the deposition of CSS‐Sb_2_S_3_ thin films with the properties (morphology, orientation, *etc*.) close to that of the CBD processed counterpart.^[^ ^27^ ^]^ Further studies are needed to verify this speculation. As a result, large‐grained compact Sb_2_S_3_ films can be achieved via seed‐mediated CSS deposition. The as‐achieved Sb_2_S_3_ films with low GB density are suggested to be ideal for constructing devices with suppressed charge recombination at GBs.

**Figure 3:**
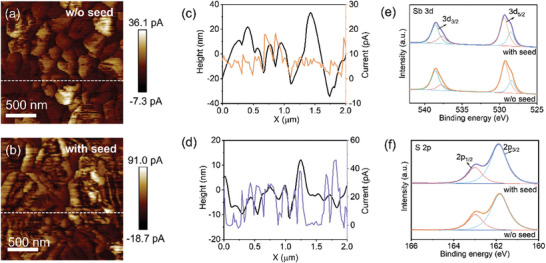
a,b) C‐AFM analysis and c,d) corresponding height profile and current of Sb_2_S_3_ films prepared without and with the seed layer. e,f) High‐resolution XPS spectra of Sb 3d, and S 2p core levels of Sb_2_S_3_ films prepared without and with the seed layer.

The 2D and 3D atomic force microscopy (AFM) topography spatial of both film samples (Figure S3, Supporting Information) reveal that the “with seed” sample shows reduced surface root‐mean‐square (RMS) roughness of ≈9.67 nm compared to the “w/o seed” sample with the roughness of ≈19.6 nm, implying that the seed layer contributes to the decrease in the surface roughness of Sb_2_S_3_ thin films. This would improve the interface quality at the back surface, thus further promoting the hole extraction from the absorber to the back electrode.^[^ ^40^, ^41^ ^]^ The conductive‐AFM (c‐AFM) images and corresponding height profile and current analysis of both film samples are given in **Figure** **3**a‐d. The intensity profiling of the surface current along the solid white line marked in Figure 3a,b is plotted in Figure 3c,d. The results reveal that both films suffer from evident current fluctuations at the GBs; compared to the “w/o seed” sample, the “with seed” sample shows more uniform and enhanced electrical conductivity across the 2 µm scale. The reduced fluctuation in the current intensity for the seed‐mediated film sample would favor the local photocurrent generation and collection, effectively suppressing the carrier recombination.^[^ ^18^ ^]^

The Raman spectra of the “w/o seed” and “with seed” Sb_2_S_3_ film samples (Figure S4, Supporting Information) indicate that both samples exhibit good crystallinity. The peak at 156 cm^−1^ is attributed to the vibration of the Sb─Sb bond, while the peaks at 193 and 238 cm^−1^ represent the asymmetric bending vibration of S─Sb─S and the stretching vibration of the Sb─S bond, respectively.^[^ ^37^ ^]^ The two main peaks at 282 and 304 cm^−1^ are attributed to asymmetric and symmetric vibrations of the Sb─S bond, respectively.^[^ ^42^, ^43^ ^]^ The Raman peaks of Sb_2_S_3_ films involve negligible shifts with the introduction of the seed layer. Figure 3e,f shows high‐resolution X‐ray photoelectron spectroscopy (XPS) spectra of Sb 3d and S 2p for Sb_2_S_3_ films, respectively. For the Sb 3d spectra, the peaks of 538.8 and 529.3 eV correspond to Sb 3d_3/2_ and Sb 3d_5/2_, respectively, and two shoulder peaks can be attributed to the oxidation state of Sb^3+^ in Sb_2_S_3_. For the S 2p spectra, two peaks observed at 161.8 and 162.9 eV are assigned to S 2p_3/2_ and S 2p_1/2_, respectively.^[^ ^5^ ^]^ The peaks of both Sb 3d and S 2p show negligible shifts, indicating that the introduction of the seed layer does not alter the elements and valence states of Sb_2_S_3_. This further confirms that the seed layer mainly mediates the grain growth and reduces the defects during the film growth.

**Figure 4:**
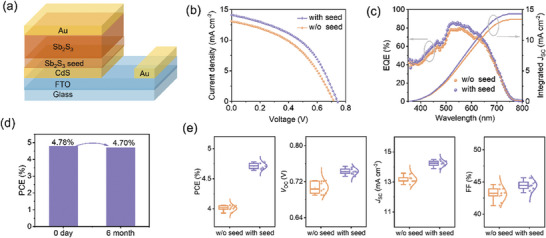
a) Schematic illustration of the superstrate planar Sb_2_S_3_ solar cell with the configuration of FTO/CdS/Sb_2_S_3_/Au. b) *J–V* curves and c) EQE spectra of Sb_2_S_3_ solar cells prepared without and with the seed layer. d) Device stability of Sb_2_S_3_ solar cells prepared with the seed layer. e) Statistics of the performance parameters (i.e., PCE, *V*_OC_, *J*_SC_, and FF) of Sb_2_S_3_ solar cells prepared without and with the seed layer.

**Figure** **4**a schematically illustrates the typical superstrate planar solar cells with the device configuration of FTO/CdS/Sb_2_S_3_/Au. The current density–voltage (*J–V*) curves of Sb_2_S_3_ thin film solar cells based on the “w/o seed” and “with seed” samples, are shown in Figure 4b, and the specific photovoltaic parameters, including *V*_OC_, *J*_SC_, FF, and PCE, are summarized in **Table** **1**. As shown, the “w/o seed” device yields a *V*_OC_ of 0.716 V, a *J*_SC_ of 13.05 mA cm^−2^, an FF of 43.31%, and a PCE of 4.05%; the “with seed” device delivers a *V*_OC_ of 0.743 V, a *J*_SC_ of 14.12 mA cm^−2^, an FF of 45.62%, and a PCE of 4.78%. That is, the incorporation of the seed layer results in relative PCE improvements of ≈18%. The average PCE of the “with seed” device (4.71%) is also higher than that of the “w/o seed” device (4.00%). Especially, the *V*_OC_ of the “with seed” device we obtained is 0.755 V, which involves a significant increase in contrast to the “w/o seed” device. Table S1 (Supporting Information) summarizes the device structure and photovoltaic parameters of previously reported Sb_2_S_3_ solar cells with competitive PCEs and well‐developed *V*_OC_ prepared by physical deposition methods, including CSS, RTE, VTD, etc. To the best of our knowledge, the obtained *V*_OC_ of 0.755 V is the highest value reported thus far for evaporation‐ and sublimation‐ processed Sb_2_S_3_ solar cells. Regarding the great influence of the GB density and film orientation on the performance of Sb_2_S_3_ solar cells, the enhanced performance of “with seed” devices should be attributed to the successful balance of the reduced GB density and more pronounced [hk0] orientations for the seed‐mediated Sb_2_S_3_ films, based on the structural and morphology characterization. The external quantum efficiency (EQE) spectra (Figure 4c) reveal that both devices show broadband photoelectric response over the 350–750 nm wavelength range, consistent with the absorption spectra of Sb_2_S_3_ films (Figure S5, Supporting Information). The “with seed” device affords higher EQE values than that of the “w/o seed” device, with the peak value exceeding 85.7% at 545 nm. The appreciable EQE enhancement observed in the long‐wavelength (520–650 nm) region indicates that the incorporation of the seed layer leads to more efficient transport and collection of the photogenerated carriers and thus reduces the charge‐carrier recombination.^[^ ^8^ ^]^ The integrated *J*_SC_ values obtained from the EQE spectra for the “w/o seed” and “with seed” devices are 13.45 and 14.39 mA cm^−2^, respectively, agreeing with *J*_SC_ values achieved from *J–V* curves. Moreover, the obtained planar Sb_2_S_3_ solar cells exhibit superior device stability. As shown in Figure 4d, the best‐performing unencapsulated “with seed” devices maintain ≈98.3% of the initial efficiency after 6 months of storage in an electronic cabinet (15% relative humidity, room temperature). The statistics of photovoltaic parameters of the “w/o seed” and “with seed” devices, each type based on 10 devices, are presented in the box charts of Figure 4e. As shown, both types of devices demonstrate excellent reproducibility, highlighting the superiority of CSS for fabricating thin film solar cells.

**Table 1:** Photovoltaic performance parameters of Sb_2_S_3_ solar cells prepared without and with the seed layer, measured under one‐sun (AM 1.5G, 100 mW cm^−2^) illumination. The values out of parentheses are the parameters of best‐performing devices for each group, whereas in parentheses are in the data format of average±standard deviation.

Device	*V*_OC_ [V]	*J*_SC_ [mA cm^−2^]	FF [%]	PCE [%]
w/o seed	0.716 (0.707 ± 0.012)	13.05 (13.14 ± 0.30)	43.31 (43.09 ± 1.05)	4.05 (4.00 ± 0.05)
with seed	0.743 (0.743 ± 0.007)	14.12 (14.23 ± 0.19)	45.62 (44.54 ± 0.82)	4.78 (4.71 ± 0.05)

**Figure 5:**
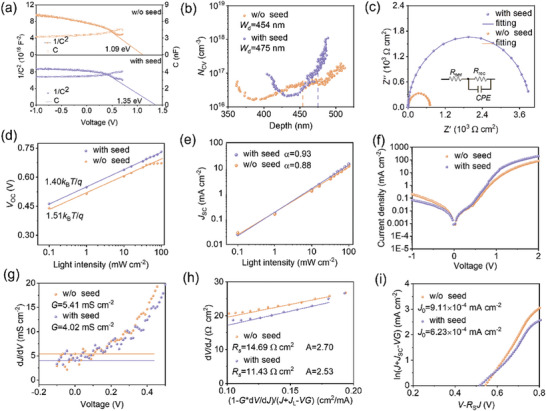
a) *C–V* and corresponding Mott‐Schottky (1/*C* ^2^∼*V*) plots of the “w/o seed” and “with seed” Sb_2_S_3_ solar cells measured in the dark, and b) logarithmic representation of a *C–V* derived carrier density profiles. c) Nyquist plots for the “w/o seed” and “with seed” devices measured in the dark (the inset shows the equivalent circuit used for fitting). d, e) The dependence of *V*_OC_ and *J*_SC_ on the light intensity for the “w/o seed” and “with seed” devices. f) Dark *J–V* curves of the “w/o seed” and “with seed” devices. Calculation of characteristic parameters in the equivalent circuit of solar cells based on *J–V* curves measured under one‐sun illumination: g) shunt conductance *G*, h) series resistance *R*_S_ and ideality factor *A*, i) reverse saturation current density *J*_0_.

**Figure** **5**a gives the capacitance–voltage (*C*–*V*) and corresponding Mott–Schottky (1/*C* ^2^∼*V*) plots of “w/o seed” and “with seed” devices measured in the dark. As shown, with an increase in the applied bias, the capacitance increases slowly at a negative bias, while it increases rapidly at the positive bias as a result of the evolution of the depletion width. The abrupt p‐n junction capacitance can be described approximately using a parallel‐plate capacitor model, in which the relationship between capacitance and bias voltage is expressed as Equation (1):^[^ ^44^ ^]^

(1) $$1/C^{2}=2\left(V_{\mathrm{bi}}-V\right)/q\varepsilon_{0}\varepsilon_{r}A^{2}N_{\mathrm{CV}}$$

where *q* is the elementary charge (1.60 × 10^−19^C), *ε*_0_ is the vacuum permittivity (8.85 × 10^−12^ F m^−1^), *ε*_r_ represents the relative permittivity of Sb_2_S_3_ (31), *A* is the active area of devices (0.06 cm^2^), *V*_bi_ is the built‐in potential, and *N*_CV_ is the charge‐carrier density.^[^ ^45^ ^]^ According to this equation, *V*_bi_ and *N*_CV_ for Sb_2_S_3_ films can be estimated. The *V*_bi_ of the “with seed” device is calculated to be 1.35 V, larger than that of the “w/o seed” device (1.09 V). The increase in the *V*_bi_ for the “with seed” device would enhance the band bending at the heterointerface and is more conducive to the efficient carrier extraction and transport in solar cells, contributing to the enhanced *V*_OC_. This trend is consistent with the *V*_OC_ values from the *J–V* curves. The *N*_CV_ profile as a function of the depth of the junction can be calculated based on the transformation of Equation (1):$N_{\mathrm{CV}}\left(D\right)=\frac{2}{q\varepsilon_{0}\varepsilon_{r}A^{2}}{\left[\frac{{\mathrm{dC})}^{-2}{\left(V\right)}^{-2}}{\mathrm{dV}}\right]}^{-1}$, where *D* is the depth of the junction, which can be obtained from *D* = ε *A/C*.^[^ ^46^ ^]^ Figure 5b shows the *C*–*V* doping profiling for both devices, where the “with seed” device delivers the wider depletion region (475 nm) compared to that of the “w/o seed” device (454 nm), benefitting the carrier generation and extraction for enhanced device performance. Moreover, the estimated *N*_CV_ of the “with seed” device (2.24 × 10^17^ cm^−3^) at 0 V bias is higher than that of the “w/o seed” device (4.74 × 10^16^ cm^−3^).

The impedance spectroscopy was further conducted to explore the charge transfer and recombination dynamics in Sb_2_S_3_ solar cells.^[^ ^47^, ^48^ ^]^ Figure 5c presents the Nyquist plots of the impedance spectra measured under dark conditions, and corresponding fitting results based on the equivalent circuit model given in the inset are summarized in Table S2 (Supporting Information).^[^ ^48^ ^]^ *R*_s_ represents the internal resistance of devices, while *R*_rec_ corresponds to the recombination resistance at the CdS/Sb_2_S_3_ interface. CPE consists of CPE‐T and CPE‐P which account for interface and ideal capacitance, respectively. The “with seed” device shows a larger recombination resistance at the CdS/Sb_2_S_3_ interface (*R*_rec_ = 62.44 kΩ cm^−2^) than that of the control device (*R*_rec_ = 11.61 kΩ cm^−2^). An increased recombination resistance would contribute to the suppression of charge recombination for enhanced charge collection in photovoltaic devices. The light intensity‐dependent *J–V* measurements were also performed to assess the trap‐assisted recombination loss mechanisms in devices. Figure 5d,e shows the dependences of *V*_OC_ and *J*_SC_ on the light intensity ranging from 0.1 to 100 mW cm^−2^ for the “w/o seed” and “with seed” Sb_2_S_3_ solar cells. The semi‐logarithmic plots of the relationship between *V*_OC_ (or *J*_SC_) and light intensity can be described by Equations (2) and (3):^[^ ^48^, ^49^ ^]^

(2) $$V_{\mathrm{OC}}\propto \left(nk_{B}T/q\right)\;\ln\left(I\right)$$

(3) $$J_{\mathrm{SC}}\propto I^{\alpha }$$

where *I* is the light intensity, *k*_B_ is the Boltzmann constant, *T* represents the absolute temperature, *q* is the elementary charge, and *n* and *α* values reflect the level of charge recombination. *n* equals 1 when the recombination is entirely radiative, and a higher *n* indicates more serious defect‐assisted Shockley‐Read‐Hall (SRH) recombination.^[^ ^50^ ^]^ As shown, the “with seed” device shows a smaller *n* of 1.40 than that of 1.51 for the “w/o seed” device. *α* should be close to 1 when the device is free of space‐charge limited current.^[^ ^8^ ^]^ Due to the space charge effect, *α* is typically less than 1. The “with seed” device has a larger *α* of 0.93 compared to the “w/o seed” device of 0.88. These results reveal that the incorporation of the seed layer leads to a reduction in non‐radiative charge recombination. The *J–V* curves of the “w/o seed” and “with seed” devices measured in the dark are shown in Figure 5f. It is revealed that both devices perform well as diodes and offer a good current rectification. The rectification ratios (herein defined as *J*_V = 0.5_/*J*_V = −0.5_) are estimated to be 1.04 and 2.15 for the “w/o seed” and “with seed” devices, respectively. The high rectification ratio implies reduced current leakage, which contributes to the decrease of the shunt resistance in the devices.^[^ ^48^ ^]^ Therefore, dark *J‐V* measurements reveal that the incorporation of the seed layer results in the suppression of current leakage, and is beneficial to the enhancement of *J*_SC_ and FF.^[^ ^42^ ^]^

We further studied the device physics by exploring parameters, including the shunt conductance (*G*), the series resistance (*R*_s_), the reverse saturation current density (*J*_0_), and the diode ideality factor (*A*), based on the analysis of the *J–V* curves measured under one‐sun illumination, as given in Figure 5g,i. According to the abrupt junction *J–V* equation as expressed in Equation (4), and its formula manipulation given in Equations (5)–(7), the characteristic parameters of the equivalent circuit model of solar cells can be extracted as follows:^[^ ^5^ ^]^

(4) $$J=J_{0}\exp\left[\frac{q}{Ak_{B}T}\left(V-JR_{S}\right)\right]+GV-J_{L}$$

(5) $$\frac{\mathrm{dJ}}{\mathrm{dV}}=\frac{q}{Ak_{B}T}\times J_{0}\exp\left[\frac{q}{Ak_{B}T}\left(V-JR_{S}\right)\right]+G$$

(6) $$\frac{\mathrm{dV}}{\mathrm{dJ}}=R_{S}+\frac{Ak_{B}T}{q}\times \frac{1-G\frac{dV}{dJ}}{J+J_{L}-\mathrm{GV}}$$

(7) $$\ln\left(J+J_{L}-GV\right)=\frac{q}{Ak_{B}T}\times \left(V-R_{S}J\right)+\ln J_{0}$$

where *q* is the electron charge, *k*_B_ is Boltzmann constant, and *J*_L_ is the photocurrent density which approximately equals the current density of the device at 0 V. The “with seed” device shows the reduced *G* (4.02 mS cm^−2^) and *R*_s_ (11.43 Ω cm^2^) compared to the “w/o seed” device (*G* = 5.41 mS cm^−2^, *R*_s_ = 14.69 Ω cm^2^), indicating that the carrier extraction in devices is improved with the incorporation of the seed layer.^[^ ^7^ ^]^ The *A* value for the “with seed” device is 2.53, smaller than that of 2.70 for the “w/o seed” device. Since the ideality factor *A* is an important parameter to evaluate the quality of the P‐N heterojunction, the decrease in *A* reflects the passivation of defects in absorber films, which might be associated with the reduction in GB density.^[^ ^5^ ^]^ In addition, the “with seed” device has a much lower *J*_0_ (6.23 × 10^−4^ mA cm^−2^) compared to that of the “w/o seed” device (9.11 × 10^−4^ mA cm^−2^). The reduced *J*_0_ implies the suppression of defect‐induced charge recombination, which is suggested to enhance the *V*_OC_ of devices.^[^ ^8^ ^]^ The analysis of these characteristic parameters reveals that the heterojunction quality is improved in the devices with the seed layer, thereby facilitating charge separation and transport for enhanced device performance.

**Figure 6:**
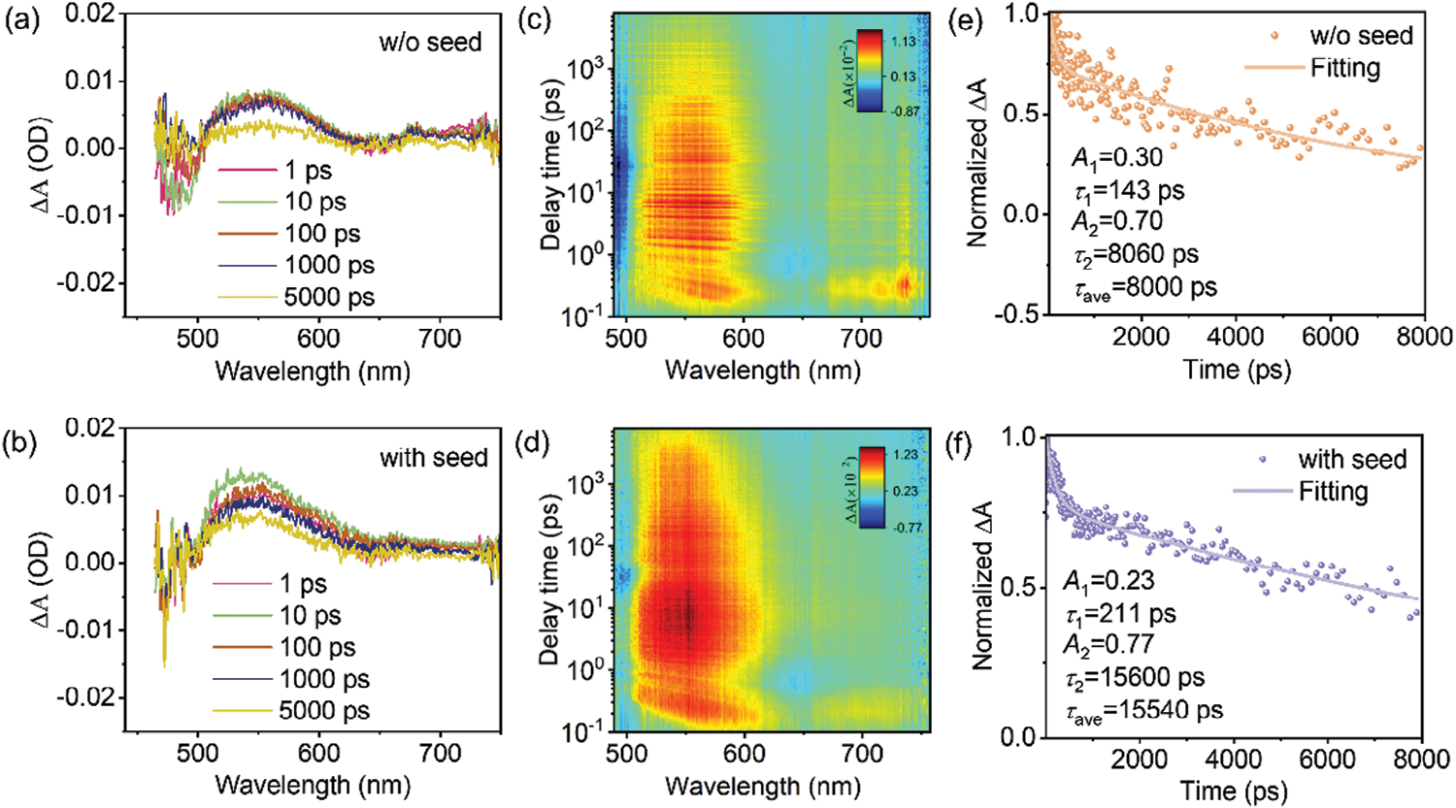
a,b) Transient absorption (TA) spectra obtained at 1, 10, 100, 1000, and 5000 ps pump‐probe delay for Sb_2_S_3_ thin films prepared without and with the seed layer. The excitation source is a pulsed laser with an excitation wavelength of 400 nm and a repetition frequency of 1000 Hz. c,d) 2D TA spectroscopy pseudo‐color images of Sb_2_S_3_ thin films. e,f) Transient kinetic decays (scatter) and corresponding biexponential curve fittings (solid line) monitored at 554 nm of Sb_2_S_3_ thin films. Δ*A* is defined as the change in the absorption of the sample before and after pumping.

We further employed ultrafast transient absorption (TA) spectroscopy to evaluate the charge‐carrier transport dynamics in Sb_2_S_3_ films. As shown in **Figure** **6**a,b, obvious ground state bleach (GSB) negative peaks and positive photoinduced absorption (PIA) peaks are identified in the TA spectra. Typically, the GSB peaks at 470–500  and 610–680 nm can be ascribed to the state filling of CdS and the ground state absorption of Sb_2_S_3_, respectively; the PIA peak at 510–600 nm is due to the formation of sulfide radical (S^−•^) as a result of the localization of photoexcited holes on the S atom of the Sb_2_S_3_ lattice.^[^ ^37^ ^]^ The transient dynamics can be obtained from the pseudo‐color images shown in Figure 6c,d. The transient kinetic decay (scatter) monitored at 554 nm for Sb_2_S_3_ films prepared without and with the seed layer are given in Figure 6e,f, and the curve fittings are performed using a phenomenological biexponential equation (solid line), with the results shown in the inset. As shown, the PIA peak involves a gradual decrease as a result of the decay of trapped holes, i.e., the S^−•^ species, which we here attribute to nonradiative carrier recombination in Sb_2_S_3_ films.^[^ ^5^ ^]^ The “with seed” Sb_2_S_3_ film sample delivers longer average lifetime (*τ*_ave_) values (15.54 ns) than that of the “w/o seed” sample (8.00 ns). Here *τ*_ave_ is calculated using the equation *τ*_ave_ = (*A*_1_ *τ*_1_^2^ + *A*_2_ *τ*_2_^2^)/(*A*_1_ *τ*_1_ + *A*_2_ *τ*_2_). The increase in the lifetime in “with seed” Sb_2_S_3_ sample suggests the suppression of charge‐carrier recombination at the GBs and interfaces. The prolonged lifetime of photoexcited minority hole carriers is suggested to contribute to the improvements of *V*_OC_ and PCE.

## 3 Conclusion

In this work, we developed an effective seed‐mediation strategy to tailor the grain growth of CSS‐processed Sb_2_S_3_ thin films, which enables to the achievement of high‐quality absorber films with low GB density. The reduction of GB density contributes to the suppression of nonradiative charge‐carrier recombination at the GBs and interfaces in the bulk absorber films, leading to reduced *V*_OC_ loss and enhanced PCE of Sb_2_S_3_ solar cells. The planar Sb_2_S_3_ solar cell based on the seed‐mediated CSS‐processed Sb_2_S_3_ thin film delivers a remarkable PCE of 4.78%, much higher than that of the conventional CSS‐processed device (4.05%). Particularly, a high *V*_OC_ of 0.755 V is achieved for Sb_2_S_3_ solar cells, and this is the record value reported thus far for evaporation and sublimation processed Sb_2_S_3_ devices. This modified CSS approach holds promise for advancing antimony chalcogenide solar cells in large‐scale manufacturing.

## 4 Experimental Section

### Device Fabrication

A simple superstrate device configuration (FTO/CdS/Sb_2_S_3_/Au) was used to build planar Sb_2_S_3_ solar cells. The fluorine‐doped tin oxide (FTO) transparent conductive glass (≈15 Ω sq^−1^) was employed as the substrate, on top of which the CdS buffer layer was deposited by a conventional CBD method, and the details of the deposition processes can be referred from previous works.^[^ ^5^, ^51^ ^]^ The Sb_2_S_3_ seed layer was deposited on the FTO/CdS substrate via CBD. Specifically, C_4_H_4_KO_7_Sb·0.5H_2_O (99.0%, Sinopharm) and Na_2_S_2_O_3_·5H_2_O (99.5%, Aladdin) were used as the antimony and sulfur sources. C_4_H_4_KO_7_Sb·0.5H_2_O (37.5 mm) and xNa_2_S_2_O_3_·5H_2_O (37.5 mm) were first completely dissolved in 40 mL deionized water, 1 mm sodium citrate (C_6_H_5_Na_3_O_7_) were further mixed and stirred for 2–3 min until completely dissolved. The FTO/CdS substrate was immersed into the precursor solution in a glass bottle and transferred into a thermostat water bath at 95 °C for 3 h. The as‐deposited Sb_2_S_3_ seed layer was rinsed using deionized water, followed by drying with N_2_ flow. The main Sb_2_S_3_ absorber layer was deposited using CSS. High‐purity Sb_2_S_3_ powders (99.999% purity, 80 mesh, Beijing Zhongnuo New Materials) were placed onto a lower graphite crucible, while the FTO/CdS/Sb_2_S_3_‐seed substrate was fixed in a graphite holder on the top, ≈8 mm away from Sb_2_S_3_ powders. The vacuum degree was kept at ≈1.0 Pa during the deposition process via continuous pumping. The substrate and the Sb_2_S_3_ source plate were first pre‐heated at 300 °C for 15 min. The source temperature was then raised to 540 °C within 1 min and kept at this temperature for 4 min. The substrate temperature remained at 300 °C during this process. Eventually, superstrate Sb_2_S_3_ devices were completed by thermal evaporation of 80 nm‐thick Au as the back electrode. The device fabrication process of Sb_2_S_3_ solar cells is illustrated in Figure 1.

### Characterization

XRD spectra of film samples were collected on an X'Pert PRO MPD diffractometer using a Cu *K*𝛼 X‐ray source (𝜆 = 1.5406 Å). SEM images were taken on a JEOL field emission scanning electron microscope (JSM‐6700F). AFM images were collected on a Bruker atomic force microscope (Dimension Icon), with the SCM‐PIT probe used in the c‐AFM measurements. UV‐vis absorption spectra were collected on a CARY 5000 Agilent spectrophotometer. XPS was conducted on a Thermo Scientific ESCALAB 250Xi spectrometer (Al *K*𝛼 excitation, 1486.6 eV). Raman spectra were obtained using the Raman spectrometer (LabRAM HR Evolution). Ultraviolet photoelectron spectroscopy (UPS) was performed on PHI5000 (He I excitation, 21.22 eV). *J–V* curves of solar cells were measured under one‐sun illumination (AM 1.5G, 100 mW cm^−2^) by using a Newport Oriel Sol 3A Solar Simulator, combined with a Keithley 2400 digital source meter. The active area of the devices was 0.06 cm^2^. EQE measurements were performed on a QTest Station 1000 ADI system (Crowntech. Inc.) under the DC mode, and the excitation source was a 300 W xenon lamp (CT‐XE‐300) combined with a M24‐S 1/4m monochromator. Impedance spectra were collected on a Zahner workstation (Zennium Pro.) with an applied voltage of 0.6 V and a scanning range of 1 Hz to 1 MHz under dark, and the spectra were fitted using the Z‐view software. The *C–V* curves were obtained by a Tonghui precision LCR digital bridge (TH2838, Changzhou, China) at room temperature at a frequency of 10 kHz and AC amplitude of 5 mV. The ultrafast TA spectroscopy was obtained on a Helios Ultrafast pump‐probe system, where a nondegenerate pump‐probe configuration was employed to detect the transient dynamics in the femtosecond to nanosecond region (50 fs to 7 ns) under ambient conditions. The excitation was with a 400 nm wavelength pulsed laser at a repetition rate of 1000 Hz, obtained by using a beta barium borate (BBO) crystal to double the 800 nm pulse generated on an optical parametric amplifier, and the white light continuum probe pulses were formed by 800 nm femtosecond with a 2 mm sapphire plate for the 400–800 nm wavelength range.

## Conflict of Interest

The authors declare no conflict of interest.

## Author Contributions

R.Z., W.W., and B.T. conceived of the idea for this manuscript, designed the experiments, analyzed the data, and wrote the manuscript. W.W. and B.T. carried out the experiments and device optimizations. L.W., X.M., H.W., G.T., and T.C. assisted in experiments and data analysis. All authors commented on the manuscript.

## Supporting information



Supporting Information

## Data Availability

The data that support the findings of this study are available from the corresponding author upon reasonable request.

## References

[1] Z. Zhu , Z. Lin , W. Zhai , X. Kang , J. Song , C. Lu , H. Jiang , P. Chen , X. Sun , B. Wang , Z. S. Wang , H. Peng , Adv. Mater. 2024, 36, 2304876. 10.1002/adma.202304876 37543841

[2] K. Wojciechowski , D. Forgács , ACS Energy Lett. 2022, 7, 3729.

[3] M. A. Green , E. D. Dunlop , M. Yoshita , N. Kopidakis , K. Bothe , G. Siefer , X. Hao , Prog. Photovoltaics Res. Appl. 2023, 31, 651.

[4] A. Wang , M. He , M. A. Green , K. Sun , X. Hao , Adv. Energy Mater. 2023, 13, 2203046.

[5] X. N. Liu , Z. Y. Cai , L. Wan , P. Xiao , B. Che , J. J. Yang , H. H. Niu , H. Wang , J. Zhu , Y. T. Huang , H. M. Zhu , S. J. Zelewski , T. Chen , R. L. Z. Hoye , R. Zhou , Adv. Mater. 2024, 36, 2305841. 10.1002/adma.202305841 37947249

[6] C. Zhao , C. Tan , D. H. Lien , X. Song , M. Amani , M. Hettick , H. Y. Y. Nyein , Z. Yuan , L. Li , M. C. Scott , A. Javey , Nat. Nanotechnol. 2020, 15, 53. 31844286 10.1038/s41565-019-0585-9

[7] C. Wang , D. Li , X. Mao , L. Wan , Z. Cheng , J. Zhu , R. L. Z. Hoye , R. Zhou , J. Mater. Chem. A 2023, 11, 19914.

[8] R. Zhou , X. Li , L. Wan , H. Niu , H. Wang , X. Yang , X. Wang , J. Hou , J. Xu , B. Xu , Adv. Funct. Mater. 2024, 34, 2308021.

[9] Z. Duan , X. Liang , Y. Feng , H. Ma , B. Liang , Y. Wang , S. Luo , S. Wang , R. E. I. Schropp , Y. Mai , Z. Li , Adv. Mater. 2022, 34, 202202969. 10.1002/adma.202202969 35668680

[10] Y. Zhao , S. Wang , C. Li , B. Che , X. Chen , H. Chen , R. Tang , X. Wang , G. Chen , T. Wang , Energy Environ. Sci. 2022, 15, 5118.

[11] X. Chen , B. Che , Y. Zhao , S. Wang , H. Li , J. Gong , G. Chen , T. Chen , X. Xiao , J. Li , Adv. Energy Mater. 2023, 13, 2300391.

[12] S. Hadke , M. Huang , C. Chen , Y. F. Tay , S. Chen , J. Tang , L. Wong , Chem. Rev. 2021, 122, 10170. 34878268 10.1021/acs.chemrev.1c00301

[13] H. Lei , J. Chen , Z. Tan , G. Fang , Sol. RRL 2019, 3, 1900026.

[14] R. Zhou , B. Tang , Q. Xie , W. T. Wu , L. Wan , S. J. Zelewski , J. Zhu , Appl. Phys. Lett. 2024, 124, 233903.

[15] X. Chen , H. Hu , J. Zhou , Y. Li , L. Wan , Z. Cheng , J. Chen , J. Xu , R. Zhou , Mater. Today Energy 2024, 44, 101621.

[16] E. S. Mungan , Y. Wang , S. Dongaonkar , D. R. Ely , R. E. Garcia , M. A. Alam , IEEE J. Photovoltaics 2014, 4, 954.

[17] L. Guo , B. Zhang , S. Li , Q. Zhang , M. Buettner , L. Li , X. Qian , F. Yan , APL Mater. 2019, 7, 041105.

[18] S. Rijal , D. B. Li , R. A. Awni , C. Xiao , S. S. Bista , M. K. Jamarkattel , M. J. Heben , C. S. Jiang , M. Al‐Jassim , Z. Song , Y. Yan , Adv. Funct. Mater. 2021, 32, 2110032.

[19] C. Liu , S. Wu , Y. Gao , Y. Feng , X. Wang , Y. Xie , J. Zheng , H. Zhu , Z. Li , R. E. I. Schropp , K. Shen , Y. Mai , Adv. Funct. Mater. 2022, 32, 202209601.

[20] J. Speigt , in Lange's Handbook of Chemistry, McGraw‐Hill, New York 2005.

[21] R. Krautmann , N. Spalatu , R. Josepson , R. Nedzinskas , R. Kondrotas , R. Gržibovskis , A. Vembris , M. Krunks , I. O. Acik , Sol. Energy Mater. Sol. Cells 2023, 251, 112139.

[22] S. Wang , Y. Zhao , B. Che , C. Li , X. Chen , R. Tang , J. Gong , X. Wang , G. Chen , T. Chen , J. Li , X. Xiao , Adv. Mater. 2022, 34, 2206242. 10.1002/adma.202206242 36030361

[23] L. X. Zhu , R. Liu , Z. Y. Wan , W. B. Cao , C. Dong , Y. Wang , C. Chen , J. W. Chen , F. Naveed , J. J. Kuang , L. H. Lei , L. Q. Cheng , M. T. Wang , Angew. Chem., Int. Ed. 2023, 62, 202312951. 10.1002/anie.202312951 37904667

[24] R. Kondrotas , C. Chen , J. Tang , Joule 2018, 2, 857.

[25] L. P. Guo , B. Y. Zhang , S. Li , Q. Zhang , M. Buettner , L. Li , X. F. Qian , F. Yan , APL Mater. 2019, 7, 041105.

[26] X. L. Li , F. Y. Gao , X. Y. Xiong , M. Q. Li , G. G. Zeng , B. Li , M. Ghali , Mater. Sci. Semicond. Process. 2023, 161, 107430.

[27] W. A. Dunlap‐Shohl , Y. Zhou , N. P. Padture , D. B. Mitzi , Chem. Rev. 2018, 119, 3193. 30387358 10.1021/acs.chemrev.8b00318

[28] M. Hao , T. Duan , Z. Ma , M. G. Ju , J. A. Bennett , T. Liu , P. Guo , Y. Zhou , Adv. Mater. 2023, 35, 2211155. 10.1002/adma.202211155 36688433

[29] C. Wang , Y. Zhao , T. Ma , Y. An , R. He , J. Zhu , C. Chen , S. Ren , F. Fu , D. Zhao , X. Li , Nat. Energy 2022, 7, 744.

[30] R. Zhou , J. Xu , P. Luo , L. Hu , X. Pan , J. Xu , Y. Jiang , L. Wang , Adv. Energy Mater. 2021, 11, 2101923.

[31] R. Lin , J. Xu , M. Wei , Y. Wang , Z. Qin , Z. Liu , J. Wu , K. Xiao , B. Chen , S. M. Park , G. Chen , H. R. Atapattu , K. R. Graham , J. Xu , J. Zhu , L. Li , C. Zhang , E. H. Sargent , H. Tan , Nature 2022, 603, 73. 35038717 10.1038/s41586-021-04372-8

[32] W. A. Dunlap‐Shohl , Y. Zhou , N. P. Padture , D. B. Mitzi , Chem. Rev. 2019, 119, 3193. 30387358 10.1021/acs.chemrev.8b00318

[33] M. J. Hetzer , Y. M. Strzhemechny , M. Gao , M. A. Contreras , A. Zunger , L. J. Brillson , Appl. Phys. Lett. 2005, 86, 162105.

[34] C. S. Jiang , R. Noufi , J. A. AbuShama , K. Ramanathan , H. R. Moutinho , J. Pankow , M. M. Al‐Jassim , Appl. Phys. Lett. 2004, 84, 3477.

[35] F. Ji , S. Pang , L. Zhang , Y. Zong , G. Cui , N. P. Padture , Y. Zhou , ACS Energy Lett. 2017, 2, 2727.

[36] X. Jin , Y. Fang , T. Salim , M. Feng , S. Hadke , S. W. Leow , T. C. Sum , L. H. Wong , Adv. Funct. Mater. 2020, 30, 2002887.

[37] W. Lian , C. Jiang , Y. Yin , R. Tang , G. Li , L. Zhang , B. Che , T. Chen , Nat. Commun. 2021, 12, 3260. 34059672 10.1038/s41467-021-23592-0 PMC8166839

[38] H. Deng , Y. Zeng , M. Ishaq , S. Yuan , H. Zhang , X. Yang , M. Hou , U. Farooq , J. Huang , K. Sun , R. Webster , H. Wu , Z. Chen , F. Yi , H. Song , X. Hao , J. Tang , Adv. Funct. Mater. 2019, 29, 1901720.

[39] X. N. Liu , Z. Y. Cai , L. Wan , P. Xiao , B. Che , J. J. Yang , H. H. Niu , H. Wang , J. Zhu , Y. T. Huang , H. M. Zhu , S. J. Zelewski , T. Chen , R. L. Z. Hoye , R. Zhou , Adv. Mater. 2024, 36, 2305841. 10.1002/adma.202305841 37947249

[40] L. Guo , B. Zhang , Y. Qin , D. Li , L. Li , X. Qian , F. Yan , Sol. RRL 2018, 2, 201800128.

[41] Z. Li , X. Liang , G. Li , H. Liu , H. Zhang , J. Guo , J. Chen , K. Shen , X. San , W. Yu , R. E. I. Schropp , Y. Mai , Nat. Commun. 2019, 10, 125. 30631064 10.1038/s41467-018-07903-6 PMC6328536

[42] H. Deng , S. Yuan , X. Yang , J. Zhang , J. Khan , Y. Zhao , M. Ishaq , W. Ye , Y. B. Cheng , H. Song , J. Tang , Prog. Photovoltaics Res. Appl. 2018, 26, 281.

[43] J. Han , S. Wang , J. Yang , S. Guo , Q. Cao , H. Tang , X. Pu , B. Gao , X. Li , ACS Appl. Mater. Interfaces 2019, 12, 4970. 10.1021/acsami.9b15148 31698902

[44] X. Mao , M. Bian , C. Wang , R. Zhou , L. Wan , Z. Zhang , J. Zhu , W. Chen , C. Shi , B. Xu , ACS Appl. Energy Mater. 2022, 5, 3022.

[45] H. Deng , S. Yuan , X. Yang , F. Cai , C. Hu , K. Qiao , J. Zhang , J. Tang , H. Song , Z. He , Mater. Today Energy 2017, 3, 15.

[46] L. Guo , S. Vijayaraghavan , X. Duan , H. G. Menon , J. Wall , L. Kong , S. Gupta , L. Li , F. Yan , Sol. Energy 2021, 218, 525.

[47] R. Zhou , Q. Zhang , E. Uchaker , J. Lan , M. Yin , G. Cao , J. Mater. Chem. A 2014, 2, 2517.

[48] J. Zhou , X. Wei , J. Zhu , X. Yang , H. Niu , L. Wan , P. Jiang , J. Xu , R. Zhou , G. Cao , Sci. China Mater. 2020, 63, 1151.

[49] J. Zhou , R. Zhou , J. Zhu , P. Jiang , L. Wan , H. Niu , L. Hu , X. Yang , J. Xu , B. Xu , Sol. RRL 2021, 5, 2100494.

[50] S. Chen , M. Li , Y. Zhu , X. Cai , F. Xiao , T. Ma , J. Yang , G. Shen , A. Ke , Y. Lu , W. Liang , H. Y. Hsu , C. Chen , J. Tang , H. Song , Adv. Energy Mater. 2022, 12, 202202897.

[51] S. Lu , H. Ding , J. Hu , Y. Liu , J. Zhu , R. Kondrotas , C. Chen , J. Tang , Appl. Phys. Lett. 2020, 116, 0008879.

