# Supplementary material for: Enhanced Performance of Close‐Spaced Sublimation Processed Antimony Sulfide Solar Cells via Seed‐Mediated Growth
Source: Adv Sci (Weinh). 2024 Oct 21;11(46):2409312. doi: 10.1002/advs.202409312 (PMC11633527; doi:10.1002/advs.202409312)
Supplement: Supplementary file 1 — Supporting Information [file ADVS-11-2409312-s001.docx]

Supporting Information

**Enhanced performance of close-spaced sublimation processed antimony sulfide solar cells via seed-mediated growth**

Wentao Wu,^1^ Bo Tang,^1^ Lei Wan,^1^ Xiaoli Mao,^2^ Haolin Wang,^3^ Guoqing Tong,^4^ Tao Chen,^3^ Ru Zhou*^,1^

^1^ School of Electrical Engineering and Automation, Hefei University of Technology, Hefei 230009, P. R. China

^2^ School of Physics, Hefei University of Technology, Hefei 230009, P. R. China

^3^ Hefei National Research Center for Physical Sciences at the Microscale, School of Chemistry and Materials Science, University of Science and Technology of China, Hefei 230026, P. R. China

^4^ School of Materials Science and Engineering, Hefei University of Technology, Hefei 230009, P. R. China

* Corresponding author:

[zhouru@hfut.edu.cn](mailto:zhouru@hfut.edu.cn) (Ru Zhou)


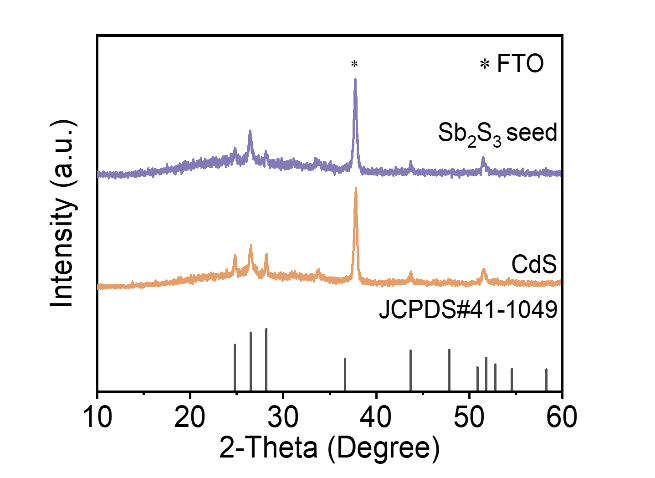


**Figure S1.** XRD patterns of the FTO/CdS substrate and the FTO/CdS/Sb_2_S_3_ seed substrate.


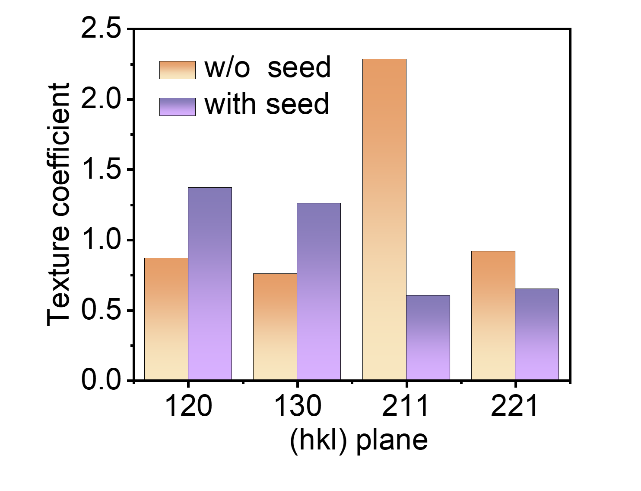


**Figure S2.** The calculated TC values of (120), (130), (211), and (221) planes. The texture coefficients (TC) of Sb_2_S_3_ thin films are calculated to evaluate the film orientation, according to the equation $TC_{(\text{hkl})}=\frac{I_{(\text{hkl})}}{I_{_{0}(\text{hkl})}}\times\left[\frac{1}{N}\sum_{i=0}^{N} \frac{I_{(\text{h}_{i}\text{k}_{i}\text{l}_{i})}}{I_{_{0}(\text{h}_{i}\text{k}_{i}\text{l}_{i})}}\right]^{-1}$, where *I*_(hkl)_ is the diffraction peak intensity of (hkl) plane for the sample, *I*_0(hkl)_ is the peak intensity of (hkl) plane obtained from the standard card, and *N* is the number of diffraction peaks used in the TC analysis.


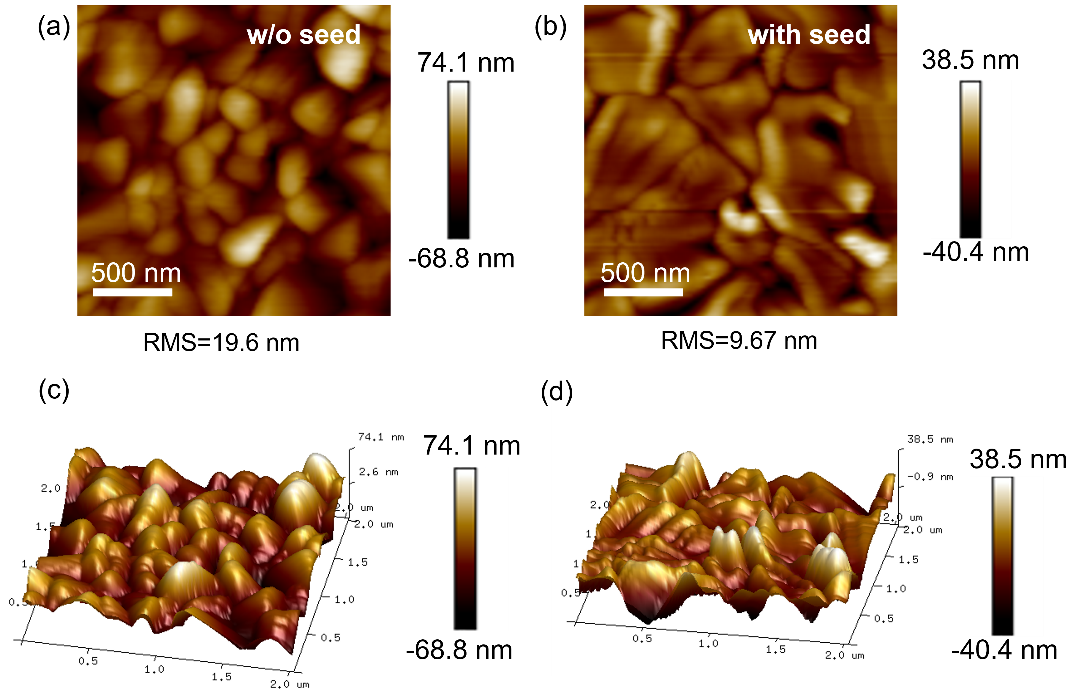


**Figure S3.** (a, b) 2D and (c, d) 3D topography spatial maps of Sb_2_S_3_ thin film samples prepared without and with the seed layer.


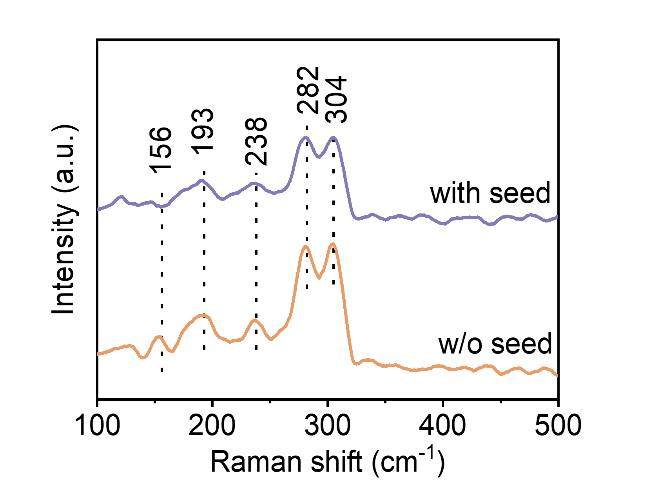


**Figure S4.** Raman analysis for Sb_2_S_3_ thin film samples prepared without and with the seed layer.

**
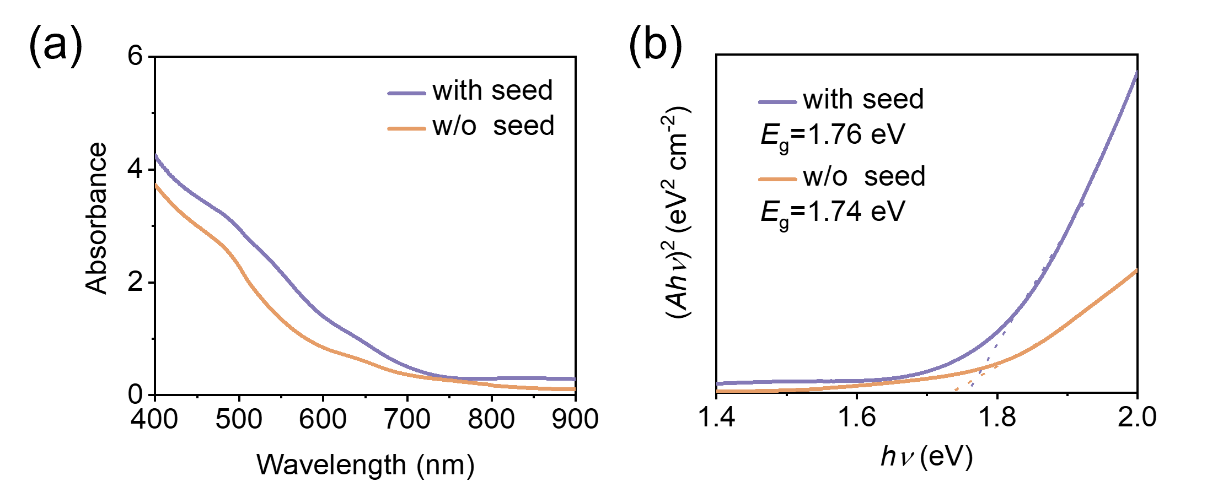
**

**Figure S5.** (a) Absorption spectra of Sb_2_S_3_ thin films deposited on the FTO/CdS substrates without and with the seed layer, and (b) corresponding Tauc plots for determining bandgaps. The bandgaps of the “w/o seed” and “with seed” film samples are determined to be 1.74 and 1.76 eV, respectively.

**Table S1**. Summary of previously reported Sb_2_S_3_ solar cells with competitive PCEs and well-developed *V*_OC_ prepared by physical deposition methods.

| Device  Structure | Method | *V*_OC_  (V) | *J*_SC_  (mA cm^-2^) | FF  (%) | PCE  (%) | Year/  Ref. |
| --- | --- | --- | --- | --- | --- | --- |
| FTO/CdS/Sb_2_S_3_/graphite/Ag | CSS | 0.660 | 13.00 | 44.65 | 3.83 | 2019^[1]^ |
| FTO/CdS/Sb_2_S_3_/Au | CSS | 0.680 | 11.50 | 48.00 | 3.80 | 2023^[2]^ |
| FTO/TiO_2_/CdS/Sb_2_S_3_/Au | CSS | 0.710 | 9.50 | 45.14 | 3.06 | 2023^[3]^ |
| ITO/CdS/ Sb_2_S_3_/Au | RTE | 0.710 | 10.8 | 47.5 | 3.5 | 2016^[4]^ |
| FTO/ TiO_2_/Sb_2_S_3_/Au | RTE | 0.57 | 10.2 | 55.3 | 3.22 | 2017^[5]^ |
| ITO/CdS/Sb_2_S_3_/Au | RTE | 0.714 | 11.39 | 51.22 | 4.17 | 2017^[6]^ |
| FTO/ TiO_2_/Sb_2_S_3_ /Au | RTE | 0.690 | 15.3 | 51.20 | 5.4 | 2019^[7]^ |
| FTO/TiO_2_/Cu- Sb_2_S_3_/Au | RTE | 0.637 | 15.15 | 47.65 | 4.61 | 2019^[8]^ |
| FTO/TiO_2_/Sb_2_S_3_/(HTL)Sb_2_Se_3_/Au | RTE | 0.680 | 15.50 | 55.40 | 5.80 | 2020^[9]^ |
| Mo/MoSe_2_/Sb_2_S_3_/CdS/ITO/Ag | RTE | 0.650 | 11.60 | 49.50 | 3.75 | 2023^[10]^ |
| ITO/CdS/Sb_2_S_3_/Au(Se/Te treatment) | RTE | 0.682 | 13.98 | 51.63 | 4.95 | 2023^[11]^ |
| FTO/ TiO_2_/Sb_2_S_3_/Au | TE | 0.588 | 10.92 | 46.77 | 3.01 | 2017^[12]^ |
| FTO/ TiO_2_/Sb_2_S_3_/Spiro/Au | TE | 0.710 | 16.11 | 50.72 | 5.80 | 2019^[13]^ |
| FTO/CdS/Sb_2_S_3_/Spiro/Au | TE | 0.720 | 15.90 | 54.30 | 6.20 | 2021^[14]^ |
| ITO/TiO_2_/Sb_2_S_3_/Au | VTD | 0.710 | 15.70 | 43.20 | 4.73 | 2020^[15]^ |
| ITO/TiO_2_/Sb_2_S_3_/Au | VTD | 0.730 | 10.90 | 56.00 | 4.50 | 2020^[16]^ |
| ITO/TiO_2_/Sb_2_S_3_/Au | VTD | 0.690 | 9.15 | 48.00 | 3.02 | 2021^[17]^ |
| ITO/TiO_2_/Sb_2_S_3_/Au | VTD | 0.660 | 11.20 | 54.23 | 4.02 | 2023^[18]^ |
| FTO/CdS/Sb_2_S_3_-seed/ Sb_2_S_3_/Au | CSS | 0.743 | 14.12 | 45.62 | 4.78 | This work |

**Table S2**. Fitting parameters for impedance spectra of Sb_2_S_3_ solar cells based without and with the Sb_2_S_3_ seed layer.

| Devices | *R*_s_ (Ω) | *R*_rec_(kΩ) | CPE-T (F) | CPE-P (F) |
| --- | --- | --- | --- | --- |
| w/o seed | 22.24 | 11.61 | 9.29 ×10^-9^ | 0.976 |
| with seed | 5.78 | 62.44 | 6.80 ×10^-9^ | 0.963 |

**References**

[1] L. Guo, B. Zhang, S. Li, Q. Zhang, M. Buettner, L. Li, X. Qian, F. Yan, *APL Mater.* **2019**, *7*, 041105.

[2] R. Krautmann, N. Spalatu, R. Josepson, R. Nedzinskas, R. Kondrotas, R. Gržibovskis, A. Vembris, M. Krunks, I. Oja Acik, *Sol. Energy Mater. Sol. Cells* **2023**, *251*, 112139.

[3] X. Li, F. Gao, X. Xiong, M. Li, G. Zeng, B. Li, M. Ghali, *Mater. Sci. Semicond. Process.* **2023**, *161*, 107430.

[4] S. Yuan, H. Deng, D. Dong, X. Yang, K. Qiao, C. Hu, H. Song, H. Song, Z. He, J. Tang, *Sol. Energy Mater. Sol. Cells* **2016**, *157*, 887.

[5] H. Deng, S. Yuan, X. Yang, F. Cai, C. Hu, K. Qiao, J. Zhang, J. Tang, H. Song, Z. He, *Mater. Today Energy* **2017**, *3*, 15.

[6] S. Yuan, H. Deng, X. Yang, C. Hu, J. Khan, W. Ye, J. Tang, H. Song, *ACS Photonics* **2017**, *4*, 2862.

[7] H. Deng, Y. Zeng, M. Ishaq, S. Yuan, H. Zhang, X. Yang, M. Hou, U. Farooq, J. Huang, K. Sun, R. Webster, H. Wu, Z. Chen, F. Yi, H. Song, X. Hao, J. Tang, *Adv. Funct. Mater.* **2019**, *29*, 1901720.

[8] M. Ishaq, H. Deng, U. Farooq, H. Zhang, X. Yang, U. A. Shah, H. Song, *Sol. RRL* **2019**, *3*, 1900305.

[9] H. Deng, S. W. Chen, M. Ishaq, Y. S. Cheng, Q. Z. Sun, X. Lin, Q. Zheng, C. X. Zhang, S. Y. Cheng, *Sol. RRL* **2022**, *6*, 2101017.

[10] H. Deng, Y. Cheng, Z. Chen, X. Lin, J. Wu, Q. Zheng, C. Zhang, S. Cheng, *Adv. Funct. Mater.* **2023**, *33*, 202212627.

[11] F. Xiao, S. W. Chen, F. G. You, T. J. Ma, C. Chen, H. Y. Hsu, H. S. Song, J. Tang, *Energy Technol.* **2023**, *11*, 202201315.

[12] X. Chen, Z. Li, H. Zhu, Y. Wang, B. Liang, J. Chen, Y. Xu, Y. Mai, *J. Mater. Chem. C* **2017**, *5*, 9421.

[13] Y. Yin, C. Wu, R. Tang, C. Jiang, G. Jiang, W. Liu, T. Chen, C. Zhu, *Sci. Bull.* **2019**, *64*, 136.

[14] W. T. Lian, C. H. Jiang, Y. W. Yin, R. F. Tang, G. Li, L. J. Zhang, B. Che, T. Chen, *Nat. Commun.* **2021**, *12*, 3260.

[15] Y. Zeng, K. Sun, J. Huang, M. P. Nielsen, F. Ji, C. Sha, S. Yuan, X. Zhang, C. Yan, X. Liu, H. Deng, Y. Lai, J. Seidel, N. Ekins-Daukes, F. Liu, H. Song, M. Green, X. Hao, *ACS Appl. Mater. Interfaces* **2020**, *12*, 22825.

[16] H. Zhang, S. Yuan, H. Deng, M. Ishaq, X. Yang, T. Hou, U. A. Shah, H. Song, J. Tang, *Prog. Photovoltaics Res. Appl.* **2020**, *28*, 823.

[17] R. Wang, Y. Wang, Y. Pan, D. Qin, G. Weng, X. Hu, J. Tao, X. Luo, S. Chen, Z. Zhu, J. Chu, H. Akiyama, *Sol. Energy* **2021**, *220*, 942.

[18] R. Wang, D. Qin, X. Ding, Q. Zhang, Y. Wang, Y. Pan, G. Weng, X. Hu, J. Tao, J. Chu, H. Akiyama, S. Chen, *J. Power Sources* **2023**, *556*, 232451.
